# Acute respiratory failure in kidney transplant recipients: a multicenter study

**DOI:** 10.1186/cc10091

**Published:** 2011-03-08

**Authors:** Emmanuel Canet, David Osman, Jérome Lambert, Christophe Guitton, Anne-Elisabeth Heng, Laurent Argaud, Kada Klouche, Georges Mourad, Christophe Legendre, Jean-François Timsit, Eric Rondeau, Maryvonne Hourmant, Antoine Durrbach, Denis Glotz, Bertrand Souweine, Benoît Schlemmer, Elie Azoulay

**Affiliations:** 1 Medical Intensive Care Unit and Biostatistics Departments, Saint-Louis Teaching Hospital, 1 avenue Claude Vellefaux, Paris F-75010, France; 2 Medical Intensive Care Unit, Bicêtre Teaching Hospital, 78 rue du Général Leclerc, Kremlin-Bicêtre F-94275, France; 3 Medical Intensive Care Unit, Hôtel-Dieu Teaching Hospital, Place Alexis Ricordeau, Nantes, 44093, France; 4 Departments of Intensive Care Medicine, Nephrology and Transplantation, Gabriel Montpied Teaching Hospital, 58 rue Montalembert, Clermont-Ferrand F-63003, France; 5 Medical Intensive Care Unit, Edouard Herriot Teaching Hospital, 5 Place d'Arsonval, Lyon, 69437, France; 6 Medical Intensive Care Unit, Nephrology and Transplantation, Lapeyronnie Teaching Hospital, 371 Avenue du doyen Gaston Giraud, Montpellier F-34295, France; 7 Department of Nephrology and Transplantation, Necker Teaching Hospital, 149 rue de Sèvres, Paris F-75743, France; 8 Medical Intensive Care Unit, A. Michallon Teaching Hospital, Avenue de Chantourne, Grenoble F-38043, France; 9 Department of Nephrology and Transplantation, Tenon Teaching Hospital, 4 Rue de la Chine, Paris F-75970, France; 10 Department of Nephrology and Transplantation, Hôtel-Dieu Teaching Hospital, Place Alexis Ricordeau, Nantes F-44093, France; 11 Nephrology and Transplantation, Bicêtre Teaching Hospital, 78 rue du Général Leclerc, Kremlin-Bicêtre F-94275, France; 12 Department of Nephrology and Transplantation, Saint-Louis Teaching Hospital, 1 avenue Claude Vellefaux, Paris F-75010, France

## Abstract

**Introduction:**

Data on pulmonary complications in renal transplant recipients are scarce. The aim of this study was to evaluate acute respiratory failure (ARF) in renal transplant recipients.

**Methods:**

We conducted a retrospective observational study in nine transplant centers of consecutive kidney transplant recipients admitted to the intensive care unit (ICU) for ARF from 2000 to 2008.

**Results:**

Of 6,819 kidney transplant recipients, 452 (6.6%) required ICU admission, including 200 admitted for ARF. Fifteen (7.5%) of these patients had combined kidney-pancreas transplantations. The most common causes of ARF were bacterial pneumonia (35.5%), cardiogenic pulmonary edema (24.5%) and extrapulmonary acute respiratory distress syndrome (ARDS) (15.5%). *Pneumocystis *pneumonia occurred in 11.5% of patients. Mechanical ventilation was used in 93 patients (46.5%), vasopressors were used in 82 patients (41%) and dialysis was administered in 104 patients (52%). Both the in-hospital and 90-day mortality rates were 22.5%. Among the 155 day 90 survivors, 115 patients (74.2%) were dialysis-free, including 75 patients (65.2%) who recovered prior renal function. Factors independently associated with in-hospital mortality were shock at admission (odds ratio (OR) 8.70, 95% confidence interval (95% CI) 3.25 to 23.29), opportunistic fungal infection (OR 7.08, 95% CI 2.32 to 21.60) and bacterial infection (OR 2.53, 95% CI 1.07 to 5.96). Five factors were independently associated with day 90 dialysis-free survival: renal Sequential Organ Failure Assessment (SOFA) score on day 1 (OR 0.68/SOFA point, 95% CI 0.52 to 0.88), bacterial infection (OR 0.43, 95% CI 0.21 to 0.90), three or four quadrants involved on chest X-ray (OR 0.44, 95% CI 0.21 to 0.91), time from hospital to ICU admission (OR 0.98/day, 95% CI 0.95 to 0.99) and oxygen flow at admission (OR 0.93/liter, 95% CI 0.86 to 0.99).

**Conclusions:**

In kidney transplant recipients, ARF is associated with high mortality and graft loss rates. Increased *Pneumocystis *and bacterial prophylaxis might improve these outcomes. Early ICU admission might prevent graft loss.

## Introduction

Kidney transplants account for about two-thirds of all solid organ transplants [1]. In patients with end-stage renal disease, kidney transplantation improves quality of life and overall survival at a lower cost than kidney dialysis [2]. Over the past two decades, the development of new immunosuppressive drugs [3] and advances in the understanding of drug management and immune modulation have reduced the incidence of acute rejection episodes and have significantly improved long-term outcomes [3-8]. The 10-year graft survival rate is now greater than 60% [1,9].

These advances have prompted increased use of kidney transplantation and substantial broadening of eligibility criteria for both donors and recipients [10-14]. It has been estimated that in 2006, 103,312 patients were living with a functional renal allograft in the United States [15]. In transplant recipients, long-term exposure to induction and maintenance immunosuppressive therapy used to prevent graft rejection carries a risk of infection, cancer and drug-related toxicities [16-19]. High-dose immunosuppressive therapy for acute rejection episodes significantly increases these life-threatening complications [16,19,20]. Furthermore, in addition to long history of chronic renal disease and dialysis, kidney transplant recipients often have severe comorbidities (for example, cardiovascular disease and diabetes) that are associated with specific immune deficiencies [2]. This combination of problems leads to complications, many of which involve the lungs [21,22]. In particular, renal transplant recipients may be at increased risk for acute lung injury (ALI) and acute respiratory distress syndrome (ARDS), most notably in the event of graft failure or antilymphocyte globulin therapy for rejection [23]. Moreover, opportunistic pneumonia is among the leading causes of death in kidney transplant recipients [24,25]. Although acute respiratory failure (ARF) compromises short- and long-term outcomes [22], few studies have assessed the need for intensive care unit (ICU) management in kidney transplant recipients with ARF.

The objective of this study was to identify determinants of survival and graft function in kidney transplant recipients admitted to the ICU for ARF. We assessed in-hospital mortality and graft function 3 months after ICU discharge [9,26].

## Materials and methods

The ethics committee of the French Society for Critical Care approved this retrospective noninterventional study and waived the need for informed consent. The study was carried out in eight medical ICUs that admit patients from nine transplant centers.

All adult recipients of a kidney or combined kidney and pancreas transplant admitted to the ICU between 1 January 2000 and 1 August 2008 were screened. Among them, we included those admitted for ARF, defined as severe dyspnea at rest, respiratory rate greater than 30 breaths per minute or clinical signs of respiratory distress and oxygen saturation less than 92% or partial pressure of oxygen in arterial blood less than 60 mmHg on room air [27].

The data reported in Tables 1, 2 and 3 were abstracted from the patients' medical charts. Life-sustaining treatments (that is, noninvasive or invasive mechanical ventilation, renal replacement therapy, vasopressors) were instituted at the discretion of the attending physicians. Criteria for noninvasive and endotracheal mechanical ventilation were also determined by the discretion of the attending physicians.

**Table 1:** Patient characteristics^a^

Demographics	All patients (*N *= 200)	Hospital survivors (*n *= 155)	Hospital deaths (*n *= 45)	*P *value
Median age, yr (25th to 75th percentile)	56 (46 to 65)	55 (44 to 64)	61 (52 to 67)	0.06
Male sex, *n *(%)	123 (61.5)	97 (62.6)	26 (57.8)	0.60
Comorbidities, *n *(%)				
Hypertension	164 (82.8)	130 (85)	35 (75.7)	0.18
Heart failure	93 (46.7)	73 (47.4)	20 (44.4)	0.74
Diabetes mellitus	55 (27.6)	41 (26.6)	14 (31.1)	0.57
Causes of end-stage renal disease, *n *(%)				0.7
Glomerulonephritis	52 (26)	41 (26.5)	11 (24.4)	
Diabetes mellitus	29 (14.5)	21 (13.5)	8 (17.8)	
Nephroangiosclerosis	24 (12)	19 (12.3)	5 (11.1)	
Polycystic kidney disease	20 (10)	13 (8.4)	7 (15.6)	
Uropathy	14 (7)	12 (7.7)	2 (4.4)	
Other or undetermined	61 (30.5)	49 (31.6)	12 (26.7)	
Characteristics of the transplantation, *n *(%)				0.28
First kidney allograft	147 (73.5)	116 (74.8)	31 (68.9)	
Kidney retransplantation	38 (19)	26 (16.8)	12 (26.7)	
Combined kidney-pancreas	15 (7.5)	13 (8.4)	2 (4.4)	
Cadaver/living donor	190/8 (96/4)	148/7 (95.5/4.5)	44/1 (97.8/2.2)	0.69
Immunosuppressive regimen, *n *(%)				
Cyclosporine	98 (50)	78 (51.3)	20 (45.5)	0.61
Tacrolimus	71 (36)	54 (35.3)	17 (38.6)	0.72
Mycophenolate mofetil	139 (71.6)	110 (72.8)	29 (67.4)	0.57
Sirolimus	25 (12.8)	21 (13.9)	4 (9.1)	0.61
Azathioprine	24 (12.2)	17 (11.2)	7 (15.9)	0.44
Steroids	170 (86.7)	132 (86.8)	38 (86.4)	> 0.99
Acute rejection, *n *(%)	43 (21.5)	34 (21.9)	9 (20)	0.84
Cytomegalovirus disease, *n *(%)	37 (19.4)	27 (18.4)	10 (22.7)	0.52

**Table 2:** Characteristics of acute respiratory failure^a^

Patient characteristics	All patients (*N *= 200)	Hospital survivors (*n *= 155)	Hospital deaths (*n *= 45)	*P *value
Median time from transplantation to ICU admission, months (25th to 75th percentile)	17 (3 to 67.3)	17 (2 to 65)	15 (3 to 98)	0.69
Median time from acute rejection to ICU admission, months (25th to 75th percentile) (*n *= 43 patients)	9.6 (2.8 to 23.8)	16.2 (3.6 to 40.8)	2.4 (0.4 to 7.2)	0.026
Median time from dyspnea onset to ICU admission, days (25th to 75th percentile)	2 (1 to 6)	2 (1 to 6)	2 (0 to 7)	0.97
Median time from hospital to ICU admission, days (25th to 75th percentile)	3 (0 to 10)	2 (0 to 9)	3 (0 to 13)	0.69
Median body temperature at ICU admission (25th to 75th percentile)	38.5°C (37.2°C to 39.1°C)	38.5°C (37.2°C to 39.1°C)	38.5°C (37.2°C to 39.0°C)	0.57
PaO_2_/FiO_2 _ratio, *n *(%)				0.14
> 300	19 (10.9)	18 (13)	1 (3)	
200-300	47 (26.9)	36 (27)	11 (28)	
≤ 200	109 (62.3)	81 (60)	28 (70)	
ICU admission directly from the emergency room, *n *(%)	61 (30.5)	46 (30)	15 (33)	0.71
Oxygen flow (L/minute) at ICU admission (25th to 75th percentile)	10 (6 to 15)	8 (5 to 15)	15 (6 to 15)	0.041
Serum creatinine (μM/L) at ICU admission (25th to 75th percentile)	250 (156 - 382)	255 (160 - 393)	240 (150 - 332)	0.23
Shock at ICU admission, *n *(%)	69 (34.8)	40 (26)	29 (64)	< 0.0001
Need for life-sustaining treatments throughout ICU stay, *n *(%)				< 0.0001
Respiratory support				
Oxygen only	77 (38.5)	75 (48)	2 (4)	
NIV	30 (15)	26 (17)	4 (9)	
NIV followed by invasive mechanical ventilation	34 (17)	23 (15)	11 (24)	
First-line invasive mechanical ventilation	59 (29.5)	31 (20)	28 (62)	
Vasopressors	82 (41)	42 (27)	40 (89)	< 0.0001
Renal replacement therapy	104 (52)	70 (45)	34 (76)	0.0006
Median SOFA score, day 1	7 (5 to 10)	6 (4 to 8)	11 (7 to 14)	< 0.0001
Median SOFA score, day 2	6 (4 to 10)	5 (4 to 7)	12 (7 to 15)	< 0.0001
Median SOFA score, day 3	5 (4 to 8)	5 (3 to 6)	12 (7 to 15)	< 0.0001
Median length of ICU stay, days	6 (3 to 12)	5 (3 to 10)	8 (3 to 15)	0.25
Median length of hospital stay, days	22 (13 to 41)	22 (14 to 43)	23 (8 to 40)	0.27

^a^ICU, intensive care unit; PaO_2_/FiO_2 _ratio, ratio of partial pressure of arterial oxygen to fraction of inspired oxygen; NIV, noninvasive mechanical ventilation; SOFA score, Sequential Organ Failure Assessment score.

**Table 3:** Characteristics of the pulmonary involvement according to the cause of acute respiratory failure^a^

Cause	Number of patients	Time (days) since respiratory symptoms onset	ARDS (PaO^2^/FiO^2 ^≤ 200) at admission	Lung infiltration ≥3 quadrants on chest X-ray	Shock at admission	Mechanical ventilation	Renal replacement therapy	Vasopressors	Hospital mortality	Day 90 dialysis-free survival
All patients	200	2 (1 - 6)	109 (62.3)		69 (34.8)	93 (47)	82 (41)	104 (52)	45 (22.5)	115 (57.5)
Bacterial infection										
Bacterial pneumonia	71	2 (0 - 44)	39 (62)	27 (40)	39 (55)	44(62)	43 (61)	39 (55)	25 (35)	33 (47)
Extrapulmonary ARDS	31	1 (0 - 20)	12 (48)	17 (57)	18 (58)	20 (65)	17 (55)	19 (61)	11 (36)	16 (52)
Cardiogenic pulmonary edema	49	1 (0 - 29)	27 (64)	41 (85)	7 (15)	14 (29)	27 (55)	11 (22)	5 (10)	29 (59)
Opportunistic fungal infection										
*Pneumocystis *pneumonia	23	10 (2 - 44)	18 (86)	20 (87)	0 (0)	12 (52)	14 (61)	9 (39)	7 (30)	11 (488)
Invasive aspergillosis or Candidemia	6	8 (0 - 45)	1 (33)	4 (67)	3 (50)	5 (83)	3 (50)	5 (83)	5 (83)	1 (17)
Viral pneumonia	6	5 (2 - 183)	2 (50)	3 (60)	0 (0)	2 (33)	1 (17)	0 (0)	0 (0)	5 (83)
Drug-related pulmonary toxicity	6	12 (1 - 183)	4 (67)	5 (83)	1 (17)	5 (83)	4 (67)	3 (50)	1 (17)	3 (50)
Other	11	1 (0 - 30)	4 (36)	3 (27)	7 (64)	4 (36)	4 (36)	5 (46)	4 (36)	6 (55)
Undetermined	25	2 (0 - 8)	14	4 (17)	5 (21)	5 (20)	6 (24)	6 (24)	2 (8)	20 (80)

^a^Data are expressed as number (%) or as median (25th to 75th percentile) for all patients and minimum-maximum for each diagnosis. A total of 203 diagnoses were made in 176 patients, and 25 patients (13%) had no diagnosis. ARDS, acute respiratory distress syndrome; PaO^2^/FiO^2 ^ratio, ratio of partial pressure of arterial oxygen to fraction of inspired oxygen.

In all patients, the diagnostic strategy implemented at the time of ICU admission included noninvasive tests (that is, echocardiography, high-resolution computed tomography, blood cultures, sputum examination, urine and serum antigens, polymerase chain reaction assay for cytomegalovirus, and *Aspergillus *antigenemia) with or without fiberoptic bronchoscopy and bronchoalveolar lavage (FO-BAL) [28,29]. The decision to perform FO-BAL was at the discretion of the attending physicians.

Disease severity was assessed using the Sequential Organ Failure Assessment (SOFA) score at admission and during the first 3 days in the ICU. Data regarding ICU and hospital lengths of stay, as well as survival status at ICU and hospital discharges and on day 90 after ICU discharge, were available for all patients. Graft survival (that is, patient survival without dialysis) 90 days after ICU discharge was also recorded for survivors.

### Statistical analysis

The statistical results are expressed as medians (25th to 75th percentiles) for quantitative variables or numbers (percentages) for qualitative variables. The characteristics of the patients and ARF episodes were compared between hospital survivors and nonsurvivors using the Wilcoxon rank-sum test or the Fisher's exact test as appropriate. To identify independent predictors of in-hospital mortality, baseline characteristics that were statistically significant and clinically relevant were included in a multivariable logistic regression model. A similar analysis was conducted to identify independent predictors of dialysis-free survival 90 days after ICU discharge. Variables entered into both models are listed in Tables 5 and 6. In both multivariable logistic regression analyses, missing values were imputed via multiple imputations by using chained equations [30]. Log-linear effects of continuous covariates were tested, calibration was tested by using the le Cessie-van Houwelingen goodness-of-fit test [31] and discrimination was assessed by the C index, which is equivalent to the area under the receiver-operating characteristic curve (AUROC) [32]. All tests were two-sided, and P < 0.05 was considered statistically significant. Analyses were performed using the R statistical package [33].

## Results

**Figure 1:**
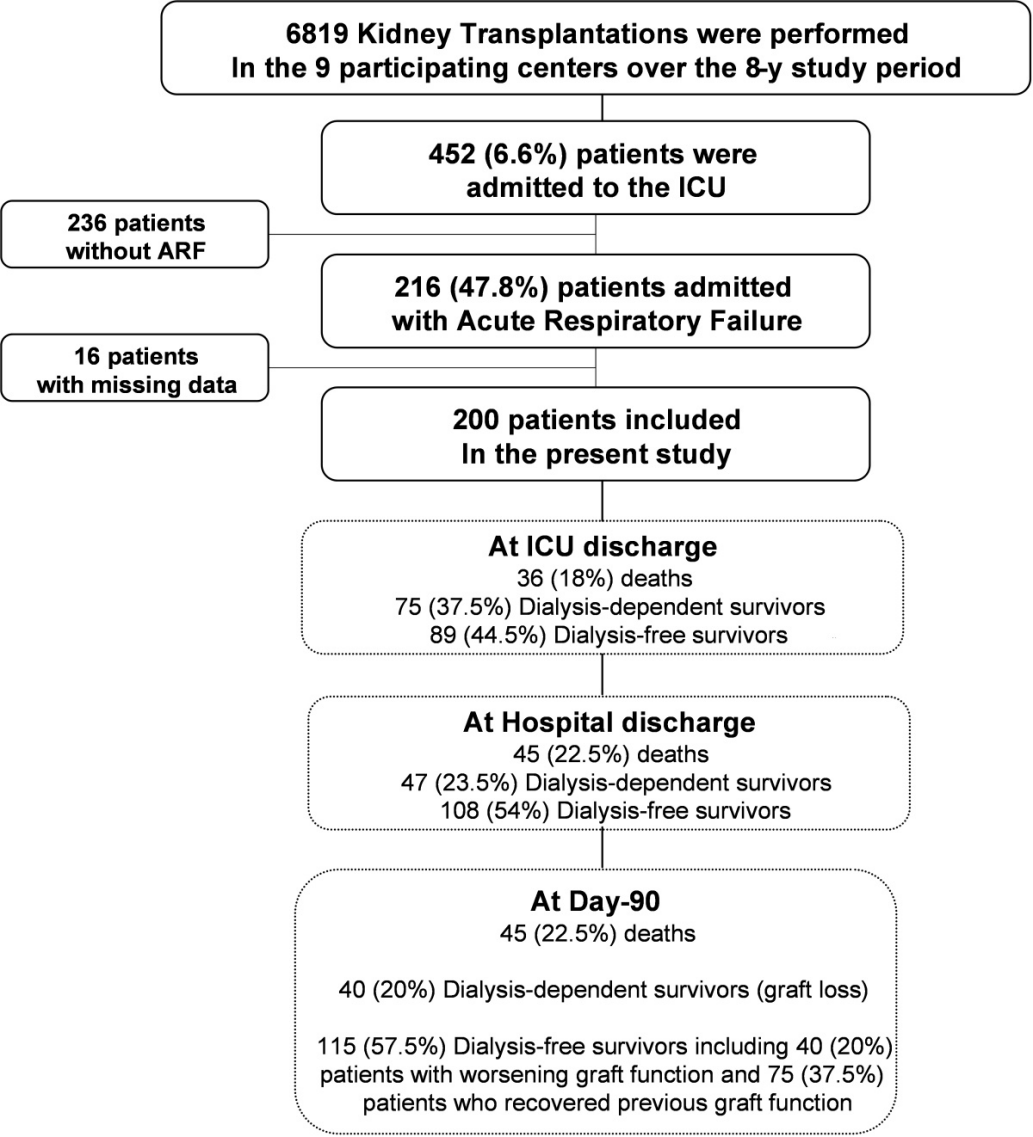
**Flowchart of the study**.

Among the 6,919 patients who received kidney allografts at the nine participating centers during the study period, 452 (6.6%) were admitted to the ICU, including 216 (47.8%) admitted for ARF. We report on the 200 patients with no missing data on day 90 (Figure 1).

Patient characteristics are reported in Table 1. Major comorbidities were hypertension (82.8%), cardiovascular disease (46.7%) and diabetes (27.6%). The three leading causes of end-stage renal disease were glomerulonephritis, diabetes mellitus and nephroangiosclerosis. Induction immunosuppressive treatment with antilymphocyte globulins or basiliximab was used in 94.1% of patients. At ICU admission, all patients were receiving immunosuppressive therapy, usually with calcineurin inhibitors (86%) combined with mycophenolate mofetil or azathioprine (83.8%) and steroids (86.7%). Forty-three patients (21.5%) had a history of acute rejection, which occurred a median of 9.6 months (interquartile range (IQR), 2.4 to 24.8) before ICU admission.

As shown in Table 2, the median time from kidney transplantation to ICU admission was 17 months (IQR, 3 to 67.3). The median time from respiratory symptom onset to ICU admission was 2 days (IQR, 1 to 6). At admission, patients were severely hypoxemic with a median of 10 L/min oxygen flow (IQR, 6 to 15). Respiratory symptoms included cough in 119 patients (59.8%), purulent sputum in 31 patients (15.5%) and chest pain in 21 patients (10.5%). Hemoptysis was noted in six patients (3%). In addition to ARF, 69 patients (34.8%) were in shock at ICU admission. Laboratory findings indicated poor graft function at ICU admission, with a median serum creatinine level of 250 μM/(IQR, 156 to 382).

FO-BAL was performed in about one-half of the patients (*n *= 113, 56.5%) and yielded the diagnosis in 45.5% of cases. Table 3 reports the clinical features and outcomes according to the cause of ARF. Bacterial pneumonia was the most common diagnosis (*n *= 71, 35.5%), with *Escherichia coli *and *Streptococcus pneumoniae *being the most often recovered pathogens, but with seven cases of methicillin-resistant S*taphylococcus aureus *and five cases of *Pseudomonas aeruginosa*), followed by cardiogenic pulmonary edema (*n *= 31, 24.5%) and ALI or ARDS related to extrapulmonary bacterial sepsis. Opportunistic fungal infections were diagnosed in 29 patients, including 23 patients with *Pneumocystis jirovecii *pneumonia, four with invasive aspergillosis, and two with Candidemia. The cause of ARF remained unknown in 25 patients (12.5%). Table 4 reports the diagnoses of ARF according to time after transplantation. In the early posttransplant period (< 1 month), cardiogenic pulmonary edema accounted for nearly one-half of the diagnoses, while opportunistic fungal infections and drug-related pulmonary toxicity were diagnosed mostly in the late posttransplant period (> 6 months).

**Table 4:** Diagnosis of acute respiratory failure according to the delay between transplantation to ICU admission^a^

Diagnosis	Number of patients	Time from transplantation to ICU admission			
		
		< 1 month	1 to 3 months	3 to 6 months	> 6 months
All patients	200	27 (14%)	30 (15%)	14 (7%)	129 (65%)
Bacterial infection					
Bacterial pneumonia	71	7 (24%)	15 (39%)	3 (19%)	46 (32%)
Extrapulmonary ARDS	31	3 (10%)	6 (15%)	4 (25%)	18 (13%)
Cardiogenic pulmonary edema	49	14 (48%)	7 (18%)	2 (13%)	26 (18%)
Opportunistic fungal infection					
*Pneumocystis *pneumonia	23	0	3 (8%)	2 (13%)	18 (13%)
Invasive aspergillosis or Candidemia	6	0	2 (5%)	2 (13%)	2 (1%)
Viral pneumonia	6	0	3 (8%)	0	3 (2%)
Drug-related pulmonary toxicity	6	0	1 (3%)	0	5 (4%)
Other	11	2 (7%)	1 (3%)	0	8 (6%)
No diagnosis	25	3 (10%)	1 (3%)	3 (19%)	18 (13%)

^a^Data are expressed as number of patients (%) and number of diagnoses (%). A total of 25 patients had no diagnosis. The 175 remaining patients had a total of 203 diagnoses. ICU, intensive care unit; ARDS, acute respiratory distress syndrome.

Noninvasive mechanical ventilation was required in 64 patients (32%) with 46.9% success, and invasive mechanical ventilation was required in 93 patients (46.5%). Vasopressors were needed in 82 patients (41%), and renal replacement therapy was administered in 104 patients (52%).

As shown in Figure 1, ICU mortality was 18% (36 deaths), and in-hospital mortality was 22.5% (45 deaths). On day 90 after ICU discharge, all 155 hospital survivors were alive, and among them, 115 patients (74.2%) were free of dialysis and 75 patients (65%) had recovered pre-ICU level of kidney function.

As reported in Table 5, independent determinants of in-hospital mortality were shock at ICU admission (odds ratio (OR) 8.70, 95% confidence interval (95% CI) 3.25 to 23.29), diagnosis of opportunistic fungal infection (OR 7.08, 95% CI 2.32 to 21.60) and diagnosis of bacterial infection (OR 2.53, 95% CI 1.07 to 5.96).

**Table 5:** Multivariable analysis: predictors of in-hospital mortality^a^

Predictor of hospital mortality	Odds ratio	95% confidence interval	*P *value
Shock at ICU admission	8.70	3.25 to 23.29	0.00002
Opportunistic fungal infection^b^	7.08	2.32 to 21.60	0.0007
Bacterial infection	2.53	1.07 to 5.96	0.034
Lung infiltration ≥3 quadrants on chest-X ray	2.50	0.98 to 6.37	0.051
Extrapulmonary ARDS	2.30	0.83 to 6.38	0.11
Oxygen flow at ICU admission (per liter)	1.05	0.97 to 1.15	0.24

^a^ICU, intensive care unit; ARDS, acute respiratory distress syndrome; area under the receiver-operating characteristic curve = 0.83; Cessie van Houwelingen goodness-of-fit test, *P *= 0.45; ^b^*Pneumocystis *pneumonia, invasive aspergillosis, or Candidemia.

Independent determinants of day 90 dialysis-free survival were worse renal SOFA score on day 1 (OR/SOFA point 0.68, 95% CI 0.52 to 0.88), diagnosis of bacterial infection (OR 0.43, 95% CI 0.21 to 0.90), lung infiltrates in three or more quadrants on chest X-ray (OR 0.44, 95% CI 0.21 to 0.91), longer time from hospital to ICU admission (OR/day 0.98, 95% CI 0.95 to 0.99) and oxygen flow at ICU admission (OR per liter 0.93, 95% CI 0.86 to 0.99) (Table 6).

**Table 6:** Multivariable analysis: predictors of day 90 dialysis-free survival^a^

Predictor variable	Odds ratio	95% confidence interval	*P *value
Renal SOFA score on day 1 (per point on SOFA scale)	0.68	0.52 to 0.88	0.004
Bacterial infection	0.43	0.21 to 0.90	0.025
Lung infiltration ≥3 quadrants on chest-X ray	0.44	0.21 to 0.91	0.027
Time from hospital to ICU admission (per day)	0.98	0.95 to 0.99	0.045
Oxygen flow at admission (per liter)	0.93	0.86 to 0.99	0.048
Shock at admission	0.61	0.29 to 1.25	0.17
Sirolimus-based immunosuppressive regimen	2.26	0.79 to 6.50	0.13

^a^Area under the receiver-operating characteristic curve = 0.77; Cessie van Houwelingen goodness-of-fit test, *P *= 0.25; SOFA score, Sequential Organ Failure Assessment score; ICU, intensive care unit.

## Discussion

We found that 6.6% of 6,819 kidney transplant recipients from nine transplant centers experienced acute illnesses requiring ICU admission and that the reason for ICU admission was ARF in about one-half of these patients. Data collected 90 days after ICU discharge showed that 22.5% of patients had died, 20% had lost their transplant and returned to dialysis, 20% had experienced deterioration in renal function and only 37.5% had recovered their pre-ICU renal function. Mortality was associated not only with the severity of the respiratory and hemodynamic manifestations but also with the cause of ARF, with bacterial and fungal pneumonia being associated with higher mortality rates. Graft loss was associated with ARF severity, bacterial infection and worse renal function at ICU admission. Importantly, later ICU admission after hospital admission was associated with a higher risk of returning to dialysis.

The ICU admission rate in our patients is in agreement with rates reported in previous studies. In a single-center study, the ICU admission rate was 6.4% [21], and other studies have found rates of up to 25% [34,35] overall and lower rates of admission for ARDS [23]. These differences may be related to differences in ICU admission criteria and in medical complications. ARF was consistently the leading reason for ICU admission in our study. Among our patients with ARF, one-third required noninvasive mechanical ventilation and nearly one-half required endotracheal ventilation.

Transplant recipients are at increased risk for infection, drug toxicities and cancer [16,20]. Infection is the leading reason for ICU admission and is significantly associated with death [36]. ARF is probably most likely to occur in kidney transplant recipients with high levels of immunosuppression, as indicated in our study by the high rate of previous acute rejection (21.5%), cytomegalovirus disease (18.5%) and retransplantation (19%). In our patients, ARF was due to infection in two-thirds of cases, and *E. coli *and *S. pneumoniae *were the most often recovered bacteria. However, the noticeable rates of resistant pathogens, such as methicillin-resistant *S. aureus *and *Pseudomonas *spp., should be borne in mind when choosing the first-line antibiotic regimen. Factors that increase the risk of resistant organisms include high-level exposure to the healthcare system during dialysis and transplantation-related assessments. Invasive fungal infections were associated with mortality in our study. Candidiasis and aspergillosis are known to be associated with very high mortality rates [24]. *P. jirovecii *pneumonia was the leading cause of opportunistic infection in our study, despite routine trimethoprim-sulfamethoxazole chemoprophylaxis as recommended [37]. However, *P. jirovecii *pneumonia occurred late after transplantation, at least 6 months after chemoprophylaxis was stopped. This important finding suggests that a longer time on chemoprophylaxis [38] may be appropriate in patients selected on the basis of a history of transplantation, acute rejection episode, pulse and chronic corticosteroid therapy, graft function and immunosuppressive regimen [25,39].

ICU mortality in our cohort was 18%, in keeping with the findings of two earlier studies (10.6% [35] and 11% [40]). The 90-day mortality rate was 22.5%, which was lower than rates reported in earlier studies [21,22,34,35,40]. Three other studies found substantially higher ICU mortality rates ranging from 36% to 58.8% [21,22,34]. These differences may be related to several factors. The studies with high mortality rates were single-center studies of small numbers of patients who had greater disease severity at ICU admission and higher SOFA scores (8.6 in the study by Klouche *et al*. [21]) or greater use of life-sustaining treatments. One study [22] included nosocomial pneumonia occurring during the ICU stay among the causes of ARF, and another [30] included mostly postsurgical patients.

In our study, only 37.5% of patients recovered their previous level of graft function, and 25.8% had to resume dialysis. In a single-center study, graft loss requiring resumption of renal replacement therapy was present at ICU discharge in 14.7% of survivors [21]. In keeping with our results, previous studies found that pre-ICU renal function was a major determinant of graft survival [26] and that ICU admission accelerated the pace of renal function decline [9]. In our study, factors associated with graft loss were worse renal SOFA score at admission, bacterial infection, involvement of more than three quadrants on the chest radiograph and longer time from hospital to ICU admission. The impact of extensive lung infiltrates in our study supports a major role for hypoxemia in loss of graft function. The deleterious impact of later ICU admission on graft survival (but not on patient survival) also deserves attention. Promptness of diagnosis and treatment is crucial to successful treatment [41]. Factors that may contribute to explaining graft loss include bacterial infection with septic shock, cardiogenic edema with a possible alteration from hypertension to hypotension and drug toxicities. Our results support early ICU referral of renal transplant recipients with ARF.

Both FO-BAL and noninvasive tests were useful in identifying the cause of ARF in our study. Immunofluorescence performed on induced sputum yielded the diagnosis of *P. jirovecii *pneumonia in three patients. Blood cultures were often positive as many patients had bacterial pneumonia and ALI or ARDS complicating extrapulmonary (mostly urinary) bacterial infection. Similarly, echocardiography was often informative. The substantial diagnostic yield of FO-BAL supports the first-line use of this procedure until more data on noninvasive tests become available. Also, given the effectiveness of noninvasive tests, we recommend adding them to the standard diagnostic strategy.

Our study has several limitations. First, we used a retrospective design. However, data collection was done specifically for this study and by the same investigator (EC) in the nine centers. Second, we included patients over an 8-year period, during which changes in treatment practices probably occurred. For instance, at ICU admission, 86.7% of our patients were on corticosteroid therapy. The use of newer immunosuppressive agents such as sirolimus, mycophenolate mofetil, T-cell and B-cell depletion and costimulatory blockade has led to a substantial number of patients being treated without long-term steroid therapy [6,19]. Third, one-fourth of our patients had cardiogenic pulmonary edema, in keeping with the high rate of cardiovascular comorbidities. Pulmonary edema does not require invasive diagnostic procedures and differs in its overall management from other causes of ARF. However, cardiogenic pulmonary edema may occur concomitantly with infection. Moreover, the aim of our study was to provide clinicians with data relevant to their everyday practice. Therefore, we included patients with ARF due to cardiogenic pulmonary edema. The strengths of our study include the multicenter design, including nine participating transplant centers, all of which had extensive experience with managing medical complications in kidney transplant recipients. Furthermore, the participating ICUs had considerable experience in managing immunocompromised patients with ARF [28,42,43].

## Conclusions

In summary, medical complications requiring ICU admission occurred in 6.6% of kidney transplant recipients, and ARF accounted for one-half of these admissions. Bacterial pneumonia, cardiogenic pulmonary edema, and ALI or ARDS related to extrapulmonary sepsis were the leading causes of ARF. *Pneumocystis *pneumonia was common and severe. By day 90 after ICU discharge, mortality was 22.5%, 20% of the patients had lost their transplant and only 37.5% of patients had recovered their pre-ICU renal function. Patient survival correlated with acute illness severity and the cause of ARF. Graft survival correlated with previous graft function, pulmonary disease severity and the cause of ARF. Our data suggest that extended chemoprophylaxis for bacterial and fungal infection and early ICU admission of patients with ARF may improve outcomes.

## Key messages

• Acute respiratory failure accounts for one-half of the ICU admissions in recipients of kidney transplantation.

• 90-day mortality is 22.5%, but a one-fourth of survivors have lost their graft.

• In the early posttransplant period (< 1 month) cardiogenic pulmonary edema accounted for one-half of the diagnoses, while opportunistic fungal infections and drug-related pulmonary toxicity were mostly diagnosed in the late posttransplant period (> 6 months).

• Fiberoptic bronchoscopy and bronchoalveolar lavage led to the diagnosis in 45.5% of cases.

• Diagnoses of bacterial or opportunistic fungal infections are associated with in-hospital mortality.

## Abbreviations

ALI: acute lung injury; ARDS: acute respiratory distress syndrome; ARF: acute respiratory failure; FO-BAL: fiberoptic bronchoscopy and bronchoalveolar lavage; ICU: intensive care unit; MV: mechanical ventilation; SOFA: Sequential Organ Failure Assessment.

## Competing interests

The authors declare that they have no competing interests.

## Authors' contributions

EC and EA conceived the study, created its design, collected the data and drafted the manuscript. JL performed the statistical analysis. DO, CG, AEH, LA, KK, GM, GL, JFT, ER, MH, AD, DG, BSo and BSc participated in collecting the data. All authors read and approved the final manuscript.
